# Hydrotalcite Intercalated siRNA: Computational Characterization of the Interlayer Environment

**DOI:** 10.3390/pharmaceutics4020296

**Published:** 2012-06-07

**Authors:** Hong Zhang, Defang Ouyang, Vinuthaa Murthy, Yunyi Wong, Zhiping Xu, Sean C. Smith

**Affiliations:** 1 Centre for Computational Molecular Science, Australian Institute for Bioengineering and Nanotechnology, The University of Queensland, Qld 4072, Brisbane, Australia; Email: hong.zhang@derm.qld.gov.au; 2 ARC Centre for Functional Nanomaterials, Australian Institute for Bioengineering and Nanotechnology, The University of Queensland, Qld 4072, Brisbane, Australia; Email: gordonxu@uq.edu.au; 3 School of Life & Health Science, Aston University, Birmingham, B4 7ET, UK; Email: d.ouyang@aston.ac.uk; 4 School of Environmental and Life Sciences, Charles Darwin University, Darwin NT 0909, Australia; Email: vinuthaa.murthy@cdu.edu.au; 5 School of Chemical & Life Sciences, Singapore Polytechnic, 500 Dover Road, Singapore 139651; Email: y.wong@sp.edu.sg; 6 Oak Ridge National Laboratory, Center for Nanophase Materials Sciences, Oak Ridge, TN 37831-6496, USA

**Keywords:** layered double hydroxide, molecular dynamics simulations, siRNA delivery, gene therapy

## Abstract

Using molecular dynamics (MD) simulations, we explore the structural and dynamical properties of siRNA within the intercalated environment of a *Mg*:*Al* 2:1 Layered Double Hydroxide (LDH) nanoparticle. An *ab initio* force field (Condensed-phase Optimized Molecular Potentials for Atomistic Simulation Studies: COMPASS) is used for the MD simulations of the hybrid organic-inorganic systems. The structure, arrangement, mobility, close contacts and hydrogen bonds associated with the intercalated RNA are examined and contrasted with those of the isolated RNA. Computed powder X-ray diffraction patterns are also compared with related LDH-DNA experiments. As a method of probing whether the intercalated environment approximates the crystalline or rather the aqueous state, we explore the stability of the principle parameters (e.g., the major groove width) that differentiate both A- and A'- crystalline forms of siRNA and contrast this with recent findings for the same siRNA simulated in water. We find the crystalline forms remain structurally distinct when intercalated, whereas this is not the case in water. Implications for the stability of hybrid LDH-RNA systems are discussed.

## 1. Introduction

Layered double hydroxides (LDHs), also known as hydrotalcites, are anionic clay materials that have received much attention due to their applications in the fields of heterogeneous catalysis [1,2], heat stabilizers [3], molecular sieves or ion exchangers [4], biosensors and halogen scavengers [5]. The general formula for LDHs is 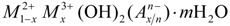, where *M*^2+^ and *M*^3+^ are divalent and trivalent metallic cations, respectively, and *A* is an anion of valence *n*. The most studied class of LDHs is Mg_6_Al_2_(OH)_16_CO_3_·4H_2_O, a popular pharmaceutical antacid talcid for ulcers. LDH structure is closely related to brucite: Mg(OH)_2_. In a brucite layer, each Mg^2+^ ion is octahedrally surrounded by six OH^−^ ions and the different octahedrons share edges to form an - two-dimensional layer. Partial replacements of Mg^2+^ ions by Al^3+^ give the “brucite-like” layers a permanent positive charge, which is balanced by negatively charged anions located in the interlayer region. This gallery also contains water molecules, hydrogen bonded to layer hydroxide and/or to the interlayer anions. Through electrostatic interactions and hydrogen bonds, the layers may be stabilized in a crystalline form. The charge density in the hydroxide layer, represented by *x* in the general formula, is normally in the range 0.2–0.33 [6]. 

The anions in the interlayer gallery are generally exchangeable and indeed anion exchange is the most widely used intercalation method. Many different kinds of anions have been successfully intercalated into LDH, including almost all of the common inorganic anions. Many organic and biomolecular anions, including carboxylated sulfates [7], benzoate [8], sulfonate [9,10], amino acids and peptides [11], as well as nucleotide phosphates and DNA chains [12], can be intercalated within the interlayers of LDHs. This unique property of intercalating organic and biomolecular molecules makes LDH a very promising delivery carrier for drug delivery and gene therapy applications [13]. Intercalation, a mechanism of encapsulation of moieties within the positive layers of the inorganic LDH, has been shown to provide sufficient protection [14] as well as to confer great potentials for controlled release properties [15]. Intercalated materials, by occupying the intergallery spacings of LDH, are often very stable in the LDH host**,** but they can also be displaced from occupation through anion exchange reactions with much more electronegative anions present in surrounding environments; this suggests a good parameter especially for site-specific targeting and release in biological systems [16]. Both the improved protection and enhanced release efficiencies are crucial aspects to consider in gene delivery systems, which undeniably suffer from many current disadvantages [17]. Several groups have performed experimental studies for DNA intercalation using layered double hydroxide. For example, Choy *et al*. have carried out extensive experiments showing that nucleotides including DNA can be intercalated into LDHs, [12] and that the metal hydroxide layers can protect DNA from catalytic and thermal degradation, making it possible for LDH-DNA intercalates to act as a molecular code system [18]. Choy *et al*. also discussed the possibility that DNA-LDH hybrid systems might act as non-viral vectors in which to transport DNA to cells for gene therapy [14]. The LDH reduces electrostatic repulsion between the negatively charged head groups of the lipid bilayer comprising the cell membrane and the anionic DNA, increasing transfection efficiency [19]. An excalation process might subsequently occur through ion exchange or degradation of the LDH in the slightly acidic cellular cytoplasm. Thus the cellular disassembly of nonviral gene delivery systems is one of the main avenues for improvement of their efficacy. The treatment of the LDH/DNA hybrids by acidic pH promotes hybrid disruption and the release of DNA from the complexes. 

While there are many more experimental studies for LDH-DNA hybrid systems, in recent years there has been growing interest in the potential for intercalating small interfering RNA (siRNA) into LDH for gene therapy applications. One example involves the introduction of small interfering RNA into neurons that potentially allows the treatment of Huntington’s (HT) disease through the RNA interference to silence the HT gene [20]. A key step in this experiment is to create a delivery system that will be able to carry the interfering RNA across the blood brain barrier and into affected neurons, for which the LDH system has been identified as a promising candidate. As is well known, the 2'-hydroxyl (2'-OH) group is the main difference between RNA and DNA and plays a fundamental role in both RNA structure and function. In general, unlike the regular conformation of DNA, such as A-, B- or Z-DNA, RNA structures are strikingly diverse, from linear duplex RNA, hairpin molecules to loop structures [21,22,23,24,25]. Among RNA double-helices, two major right-handed conformations: the 11-fold helix of A-RNA and 12-fold helix of A'-RNA have been identified experimentally in crystal structures [26,27]. The major A-RNA conformation is apparent in natural RNA polynucleotides, or when crystallized from solutions of low ionic strength, while A'-RNA is formed from higher ionic strength solutions [27]. The two forms of RNA retain the same overall helical features, however A'-RNA holds a wider major groove than that of A-RNA. The details of their structural characteristics are discussed in several texts about nucleic acidstructures [28,29,30].

The interaction of RNA with minerals such as montmorillonite and LDH are very important, and experiments have been conducted by Ferris *et al*. to show how montmorillonite can catalyze the oligomerization of suitably activated nucleotides to form strands of RNA [31]. This reaction is of importance to origins of life studies, which have yielded evidence that minerals may have played an important role in prebiotic synthesis [32]. Specifically, the RNA World hypothesis postulates that since RNA itself can act as a catalyst, in addition to carrying genetic information, the earliest forms of life were built upon RNA [33]. Deep ocean hydrothermal vents have been suggested as possible sources for precursors of pre-biological molecules, and in such environments, it has been argued that minerals including LDHs could have further concentrated the primitive molecules, eventually leading to the formation of larger organic compounds. Although deep ocean hydrothermal vents have generated particular interest as a possible source of the first life forms, there remains the question of how biopolymers such as RNA could have remained intact at the elevated temperatures and pressures around these vents. One possible explanation is that clay-like particles may have acted as structures which supported and protected nucleic acids once formed. 

In general, LDHs are polycrystalline materials and precise experimental location of interlayer anions such DNA or RNA is extremely difficult. Only rarely can sufficiently large crystals for full structural determination by conventional single-crystal X-ray diffraction be obtained. Powder X-ray diffraction (PXRD) gives some indication of the bulk structure of the material, but in general LDH intercalates are characterized by the absence of significant long range order. Reflections tend to be broad and structure-solution from powder X-ray data has been achieved *only for small inorganic guests*. Interlayer arrangements may be postulated from PXRD patterns, but are frequently little more than educated guesses based solely on the molecular dimensions of the guest. Moreover, the interlayer arrangements depend strongly on the interlayer water content of the LDH as well as the anion size. Other techniques such as FTIR have been used to characterize LDHs, but these give only limited information about the arrangement of the guests within the interlayer region. More recently, computational atomistic simulation methods have been employed to study the arrangement of the guest ions inside LDH systems [34,35,36,37,38], which are likely to provide, at the moment, the only means of knowing the details of the interlayer arrangements, given the poorly crystalline nature of the intercalates. The use of computer modelling has thus greatly augmented our present understanding of the orientation and organization of the inter-lamellar guest molecules. In this regard, molecular dynamics (MD) provides a powerful technique to probe the structure as well as the dynamic properties at a molecular level and offers a direct connection between local structural details and experimental measurements. So far several groups have performed molecular dynamics (MD) simulations of interlayer arrangements and energetics for LDHs containing organic or biomolecular anions such as terephthalate [39], cinnamate [35], carboxylic acids [40], amino acid [34] and DNA [41]. Experimentally observed parameters, such as the interlayer spacing, can be simulated successfully. In addition, close contacts and preferred orientations of the intercalates have been analyzed through the simulations. In these simulations different force fields such as modified Dreiding force field [42], CLAYFF force field [43], and CVFF force field [44] (or hybrid force field) have been used and different hydration states have been modeled. While the reliability of the simulation outcomes largely depends upon the accuracy of the force field employed, the simulations can provide significantly increased molecular-scale insights into the structural and energetic origins of the interactions of organic molecules with LDH compounds. Combined with experimental results, these computer simulations can provide a clearer and more detailed picture of the different arrangements in the interlayer of the clay systems beyond pure geometric considerations (see, e.g., [8,36]).

As elaborated above, understanding on the atomistic level both the structure and dynamics of RNA molecules intercalated within LDH layers is critical to gaining a clearer picture of their role and function as therapeutic agents in gene therapy applications, as well as their possible role in prebiotic synthesis. This challenge can in principle be addressed by a combination of experimental approaches and molecular dynamics simulations, as we did recently for a simpler LDH-sulfonate system [45]. Our previous publications have shown the experimental details of the preparation and characterization of LDH-siRNA nanoparticles [20,46]. In our experiments, the average size of LDH nanoparticles were about 100 nm and the characterization studies of the nanoparticles showed the characteristics of MgAl–LDH-type materials [46]. Based on the zeta potential values of the nanomaterials, it was estimated that over 85% of siRNA among total absorbed RNAs was intercalated into the interlays of LDH due to the electrostatic interaction between the nanomaterials and nucleic acids [20]. However, existed experimental techniques only provide very limited information about the structures of LDH-RNA nanoparticles. The main purpose of the present paper is to use computer simulation to provide insight into the structure and stability of siRNA while intercalated in layered materials, as a complement to the limited information that can be obtained from experiments. In what follows we will use molecular dynamics to extract detailed information on the structure and dynamics of RNA intercalated within Mg_2_Al-LDH. In our recent study of the dynamical properties of the A and A' crystalline forms of RNA when relaxed and equilibrated in aqueous solution, we discovered that the two forms do not appear to be structurally distinct in the aqueous environment [47]. By comparing and contrasting the intercalated A and A' forms, the present work yields new insights into the nature of the intercalated environment in contrast to the aqueous environment. 

## 2. Simulation Methods

In this section we outline the techniques used to simulate the LDH-siRNA system. We have employed a classical force field to perform geometry optimization and molecular dynamics simulations. For the complicated hybrid LDH-siRNA system, the general *ab initio* force field (Condensed-phase Optimized Molecular Potentials for Atomistic Simulation Studies: COMPASS) [48] is used for all the molecular dynamics simulations. 

### 2.1. Model Construction

The initial LDH model used in this study was built from the crystallographic structure data obtained from the refinement by means of Rietveld procedure [49]. The atomic coordinates are constructed from the previously reported crystal structure of hydrotalcite, Mg_4_Al_2_(OH)_12_CO_3_·3H_2_O. The unit cell of the original host structure is trilayer, and the space group is 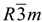 (this symmetry is the most commonly found in nature). The rhombohedral lattice parameters are *a* = 3.0460 Å, *c* = 22.772 Å, *α* = 90°, *β* = 90° and *γ* = 120°. The initial interlayer carbonate anions CO_3_^2^^−^ and water molecules were then removed, and a supercell of 24*a*× 10*a*× 2*c* was set up with lattice parameters 24*a* = 73.1042 Å, 10*b* = 30.460 Å, and 2*c* = 45.5444 Å. The three layer repeat was built with a central layer spacing of 30.363 Å and the other two layer spacing of 7.591 Å. Each hydroxide layer contains 77 Al^3+^ and 163 Mg^2+^ ions, with the latter being arranged in such a way that they are not located in adjacent octahedrals. Thus, the final *Mg:Al* ratio in the LDH layer is roughly 2:1. The simulation model thus consists of three host layers and three guest layers. 

One optimized siRNA molecule (see below for the optimization procedures) was introduced into the central LDH interlayer. The sequence of the 21 base pair siRNA is taken from the earlier study by Putral *et al*. [50] and is as follows:

   Sense          5'-GCAACAGUUACUGCGACGUUU-3'

   Antisense      3'-UUCGUUGUCAAUGACGCUGCA-5'

Both 3'-terminal UU of the RNA duplex were cut to form a canonical RNA duplex and then A-RNA and A'-RNA was generated in the NUCGEN module of AMBER. The phosphate groups of all RNA strands were unprotonated with a unit negative charge. The above sequence of the siRNA strand was chosen as it has been well studied in our latest molecular dynamics simulations [47], so a detailed understanding can be obtained of how its behavior changes while intercalated in LDH. In that study, the A form and A'-form siRNA strands were simulated in bulk water using AMBER forcefield.

**Figure 1:**
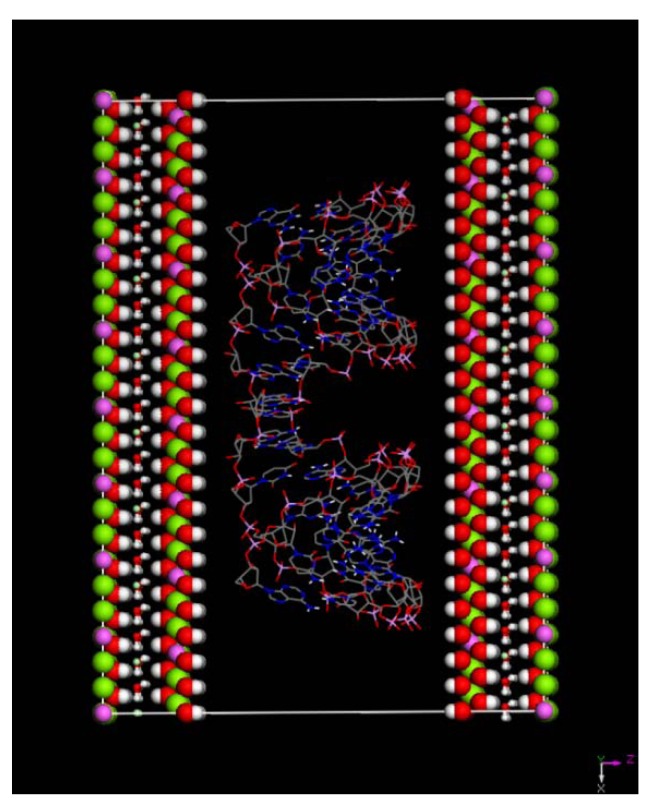
Plot of whole model system prior to relaxation for A-RNA plus Layered Double Hydroxide (LDH) system. The water molecules in the central layer have been omitted for clarity.

To minimize interactions between RNA and its periodic images we imposed a minimum distance of 6 Å between RNA and the edge of the simulation box in our systems. Since the charge of each layer is +40.425e, and one siRNA has the negative charge of 36e, totally 86 counter-ions chlorine Cl^−^ have been added into the two edge layers in order to neutralize the positive charges for the hybrid system. All three inter-layers were then inserted with 2640 water molecules in the central layer immersing siRNA, and 320 water molecules in the two edge layers in between the OH^-^ groups in the LDH layers (together with Cl^−^ anions to form a single layer). This procedure resulted in the hybrid structures containing approximately 13,781 atoms which include 3600 LDH atoms, the 1215 RNA atoms, 86 counterions (Cl^−^), and the remainder 8880 atoms were from water molecules. In Figure 1 we show the periodic cell utilized in the calculation after construction of the intercalated model, prior to relaxation (central layer water molecules omitted for clarity).

### 2.2. Minimization

The Discover Minimizer module in Material Studio (MS) version 4.4 was employed to optimize the siRNA anion first. The COMPASS force field was chosen to perform the optimization, and Smart Minimizer procedure was used for the minimization. The total number of iterations used for the convergence is 3000 for the isolated siRNA anion. Next we inserted the minimized siRNA anion into the central LDH layer to form the hybrid system. The minimization procedure for the whole LDH-siRNA systems consisted of two steps. In the first step, the central layer RNA and water molecules are allowed to relax while all other parts were fixed. The structures were subjected to 1500 steps of steepest descent minimization followed by 1500 steps of conjugate gradient minimization. In the second step, the whole hybrid system is allowed to relax including the water and Cl^−^ ions in the two edge layers and the LDH layers. The entire system was minimized by 500 steps of steepest descent minimization followed by 500 steps of conjugate gradient minimization without the restraints.

### 2.3. Molecular Dynamics Techniques

Discover in Material Studio (MS) 4.4 was employed to perform molecular dynamics simulations for studying siRNA-LDH intercalations. After energy minimization, two subsequent MD simulations were performed for both A-form and A'-form RNA intercalated into LDH. Firstly, the LDH framework and Cl^−^ ions and water molecules in the two edge layers in the super-cell were fixed while MD simulations were performed for the intercalated siRNA species and water in the central layer. Following this, the full hybrid system was relaxed for an ensuing MD simulation. The total non-bonded potential interaction energy of the simulated system consists of long-range Coulombic interactions between partial atomic charges and van der Waals interactions, computed using atom based summation method. The cut off distance is set to 9.5 Å. All the atoms including the LDH, water, chloride ions, siRNA are assigned with the COMPASS force field, excepting only the charges for the LDH part which were modified according to CLAYFF force field of Cygan *et al*. [43], because the layer charge can have a major influence on the anion packing mode in the interlayer [35]. In CLAYFF force field, we set the partial charge for Al to 1.575e, for Mg to 1.05e, for O to −0.95e and for H to 0.425e. 

Following the same protocol as our previous simulations [51], molecular dynamics simulations were performed for a constant-volume/constant-temperature ensemble (NVT) at room temperature *T* = 298 K. A time step of 1.0 fs was used, and the total simulation time was 500 ps. Temperature was maintained using Andersen thermostat with a collision ratio 1.0. Periodic boundary conditions were applied in three dimensions so that the simulation cell is effectively repeated infinitely in each direction. Analysis reveals that the equilibrium values for the crystallographic parameters and thermodynamics parameters were generally reached within the first 100 ps. All parameters were calculated using Discover module in MS 4.4 using the trajectory files generated from the 500 ps fully relaxed MD simulations. 

For the powder X-ray diffraction pattern calculations, we have employed the REFLEX module in Discover in MS 4.4. In our calculations the diffractometer range 2*θ* was set from 2° to 50° with the step size of 0.05 degree, and the radiation wavelength was set to 1.54180 Å, which matches with the experimental set up (e.g., in ref [52]). PXRD patterns of LDH-RNA systems are not as well defined as those of LDHs intercalated with simpler anionic species such as nitrate and chloride ions. Available related experimental evidence from PXRD patterns (e.g., in refs [52,53]) all points to the partial intercalation of DNA in the MgAlLDH galleries. Therefore only one out of three LDH galleries was intercalated with siRNA in this paper. 

## 3. Results

### 3.1. Simulation Results

**Figure 2:**
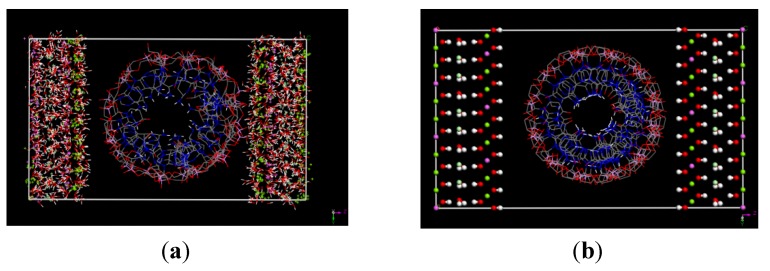
A'-RNA plus LDH structures before intercalation (**a**) and after intercalation (**b**). Cross section is in the y*z* plane. The structure in (**b**) is taken from final snapshot of the fully relaxed LDH-siRNA simulation.

The optimized structures for the gas-phase model of A-RNA and A'-RNA are shown in Figure S1. In the first stage of optimization of the intercalated system only the siRNA and water molecules in the central layer are allowed to relax, with all other parts held fixed. Figure S2 shows the minimized structure for the partially constrained hybrid systems. Following the initial constrained minimization, the hybrid LDH-siRNA systems are fully relaxed without any constraints. Figure S3 shows the unconstrained optimized structures for A-RNA and A'-RNA, respectively. After minimization, two separate 500 ps MD simulations were performed for the partially constrained (*i.e*., outer layers fixed) and the unconstrained systems using the COMPASS force field. In order to show clearly the deformations of siRNA after intercalation into LDH, in Figure 2 we show the A'-form RNA plus LDH structures before (a) and after (b) optimization as one example. In the solvated and optimized structure plotted in Figure 2b, we have suppressed most water molecules close to the RNA in order to make its structure clearer. From Figure 2b we can see that if viewed along the helical axis, the intercalated RNA has significant deformations, yet still retains the essential circular cross section of the helical structure. As suggested above, the deformations may be due to the electrostatic interactions between the RNA phosphate backbone and the neighboring two LDH surfaces. The backbone close to the LDH layers has the largest electrostatic interactions and re-arranges itself, making them closer to LDH surface. For other RNA phosphate backbone elements further away from LDH layers, the electrostatic interactions will be smaller. This re-arrangement may in fact break some of the original hydrogen bonds. It has been suggested on the basis of circular dichroism experiments that DNA decreases in length in order to optimally align itself with Al^3+^ ions in the LDH sheets, resulting in a decrease in base pairs per turn from 10.4 to 10.2 [54]. The plot in Figure 2 for our A'-RNA + LDH system suggests a little bit expansion of RNA which can be seen in the y-z directions. This RNA expansion, particularly along the *y*(*z*) axis (which is perpendicular to the helical axis) is consistent with simulations for LDH-DNA system I. [41] (Here and where relevant for the figures below, some corresponding pictures for the A'-RNA structures have been provided in the SI part). Inspection of the structures for the hybrid system as shown in Figure 2 shows that the RNA double helix orients parallel to the hydroxide layers. It is consistent with the experimental suggestion of highly disordered arrangement from the broad peaks in PXRD patterns (see below for more details). One may conjecture that the RNA structure as a whole must be able to deform within the LDH in order to allow the phosphate groups in RNA to organize themselves in the vicinity of positively charged regions of the clay. However, despite this, the RNA retains its fundamental helical structure and (as noted above) the two different crystalline forms simulated remain distinct from one another. 

**Figure 3:**
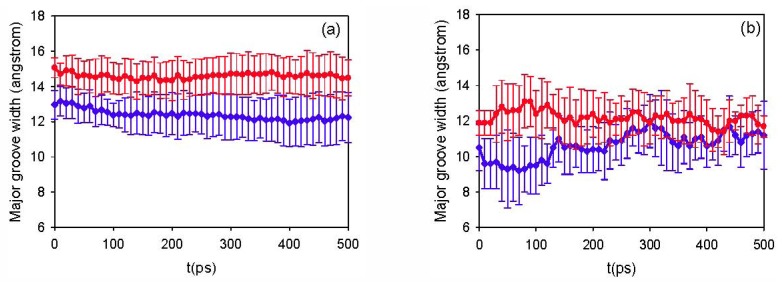
Plot of average major groove width as a function of time (standard deviation indicated by vertical bars) for siRNA in crystallized LDH layers (**a**) and in water (**b**) [47]. Blue line (with diamonds) and red line (with circles) represent the results for A-RNA and A'-RNA, respectively.

As is well known, in crystal structures [26,28,29,30,55,56], the two forms of RNA retain the same overall helical features, however A'-RNA holds a wider major groove than that of A-RNA. A comparison of the two forms of siRNA in these simulations indicates that they retain separate structural integrity as distinct stable isomers over the 500 ps timeframe. In Figure 3 we plot the average major groove width as a function of time (averaged along the length of the strand at any given time point), as this is the major structural parameter utilized to distinguish the two forms. Figure 3a corresponds to the case of siRNA when intercalated into LDH layers, whereas Figure 3b to the case of siRNA in aqueous solution [47]. From this figure we can clearly see that throughout the 500 ps simulation the major groove width for intercalated A'-RNA remains significantly larger than that of intercalated A-RNA. This is demonstrably not the case in water, however, where the two structures have already attained the same groove width by 300 ps. For the intercalated system, at no stage during the simulation do the strand-averaged values of the major groove width for A-RNA and A'-RNA converge to the same value, whereas they overlap significantly in the water-based simulations. In fact, the previous MD simulations over a longer period of 30 ns in aqueous solution showed no evidence that the A- and A'-RNA structures that seeded the separate MD simulations could be regarded as independent conformations over time [47]. Large fluctuations for the groove widths were observed over the simulation time, indicating the intrinsic flexibility of siRNA in the aqueous solution. The present 500 ps simulation time is quite short compared to the previous aqueous simulations. However, Figure 3 reveals consistent trends, as summarized above, that show no sign of abating. The average groove widths found in this intercalation study are larger than those of the previous aqueous RNA simulation. To investigate the effect of different forcefield, we simulated the siRNA in water by COMPASS forcefield in MS4.4 and the results were shown in Figure S7. Figure S-7 indicated that the structural distinctness of the two forms of RNA by COMPASS force field was preserved and less fluctuation than that in AMBER forcefield (as shown in Figure 3b). There is a little different between COMPASS forcefield and Amber forcefield in the description of nucleic acids. However, there were still more vigorous fluctuation of RNA in water (as shown in Figure S-7) than that in LDH layers (as shown in Figure 3a). Moreover, as Table S-1 shows, the self-diffusion coefficients of water and siRNA in LDH hydroxide layer are 100 times lower in all cases than the values obtained from simulations of bulk water, which also conforms to the less fluctuation of RNA in LDH layer. Thus, the intercalated RNA in LDH has less degree of dynamical flexibility in structures compared with the aqueous environment. There are two possible reasons responsible for this phenomenon: strong electrostatic interactions between LDH and RNA hinder the move of intercalated siRNA compared with more mobile in water; and/or RNA has more limited space within LDH than free aqueous solutions. 

**Figure 4:**
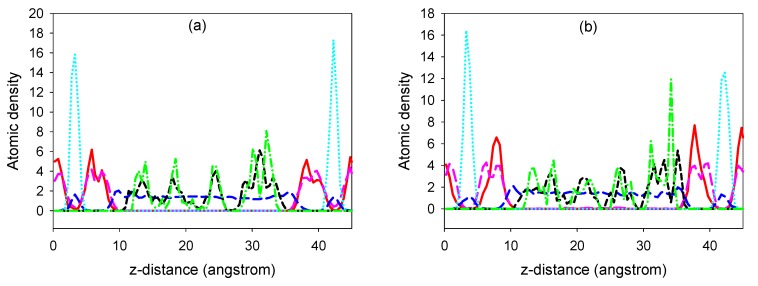
Atomic density profiles from 500 ps of fully relaxed MD simulations at 300 K for LDH + A-RNA in (**a**) and LDH + A'-RNA in (**b**). The red solid line represents Mg atoms in the LDH layer, the pink long dashed line represents the oxygen atoms in the LDH layers, the blue medium dashed line represents oxygen atoms of water, the black short dashed line represents oxygen atoms of phosphate groups in siRNA, the cyan dotted line represents Cl anions, and finally the green dash-dot line represents P atoms in siRNA.

Despite the distortions of the LDH system apparent from the fully relaxed simulations as shown in Figure 2b, broadly layered structures for the siRNA-LDH hybrid systems are still clear from concentration profile analysis. In Figure 4 we show the atomic density profiles orthogonal to LDH layers for selected atoms in the hybrid systems. These analyses are based on the trajectories from 500 ps of fully relaxed MD simulations for the LDH-siRNA hybrid system at 300 K. From this figure we can see the hybrid system still has a well defined structure, with oxygen atoms of OH in the LDH layer closest to the Mg/Al sheet (the z distance is zero for the first Mg/Al sheet in LDH layer), followed by oxygen atoms of water as well as oxygen atoms in the phosphate groups in siRNA, and finally the P atoms of phosphate groups in siRNA. The *z* distance of some oxygen atoms in water and in the phosphate groups of siRNA are actually very close, and can be regarded at the same layer in general. Our results indicate that Cl^-^ ions in the two edge LDH layers are well structured due to its sharp peaks (together with water molecules to form one single layer), followed by Mg atoms in the LDH layers, and then by P atoms in the phosphate groups of siRNA. Finally the water molecules in the central layer are less structured, albeit demonstrating some peaks in the distribution. 

### 3.2. Diffusion Coefficients

Next we report the calculated dynamical properties, e.g., the mean-square displacement (MSD) and thus the self diffusion coefficients (*D*), for the intercalated siRNA and water. The analysis can provide some useful information for the stability of the hybrid system, which is in turn very important for the efficient delivery of siRNA using LDH nano-particles as carriers. The analyses are based on the trajectories from the fully relaxed MD simulations only. 

In Figure S8 we firstly show the calculated MSD for intercalated water molecules from 500 ps of fully relaxed MD simulations at 300 K. Figure S8a corresponds to LDH + A-RNA case, while Figure S8b to LDH + A'-RNA case. The linear nature of the MSDs suggest that, for the purpose of calculating *D* for water molecules in these models, 500 ps of molecular dynamics simulations is satisfactory, although longer simulations would be more desirable for more accurate simulations. The water self-diffusion coefficient *D* for LDH + A-RNA system was calculated from the gradient of the simulated MSD with time, which is roughly 2.7 × 10^−7^ cm^2^/s at 300 K. For water in LDH + A'-RNA system, the estimated self-diffusion coefficient *D* from the gradient of the simulated MSD is 1.3 × 10^−7^ cm^2^/s at 300 K. These values for *D* are in between the simulated water self-diffusion coefficient of 4.4 × 10^−7^ cm^2^/s at 300 K for Mg_3_Al (terephthalate) LDH (with 64 water molecules), and 1.1 × 10^−7^ cm^2^/s at 300 K for Mg_2_Al (terephthalate) LDH (with 44 water molecules) [57]. As would be expected for water constrained between the LDH hydroxide layer and the siRNA, the simulated water self-diffusion coefficients are much lower in all cases than the values obtained from simulations of bulk water (1.88 × 10^−5^ cm^2^/s at 300 K, using Dreiding forcefield or TIP3P water parameters and 3.2 × 10^−5^ cm^2^/s at 300 K, using COMPASS forcefield) [58] or the experimental value obtained for the bulk water (2.3 × 10^−5^ cm^2^/s at 298 K) [59]. This is due to the geometry constraints for water in the LDH layers, thus water molecules are not as mobile as in the bulk case. This result is in agreement with the less fluctuation of intercalated RNA within LDH.

**Figure 5:**
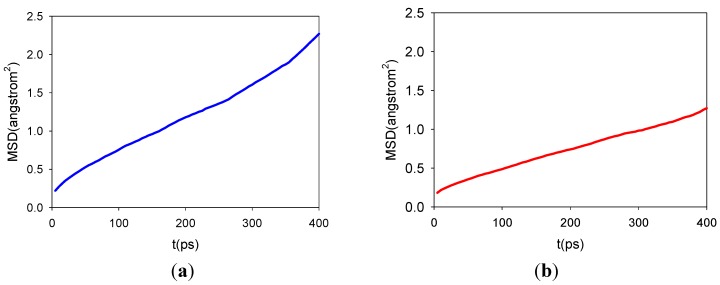
Calculated MSD for A-RNA in (**a**) and A'-RNA in (**b**) in the LDH hybrid systems from 500 ps of fully relaxed molecular dynamics (MD) simulations at 300 K.

In Figure 5 we show the calculated MSD for the intercalated siRNA from 500 ps of fully relaxed MD simulations at 300 K. Figure 5a corresponds to A-RNA and Figure 5b to A'-RNA. The A-form RNA self-diffusion coefficient *D* was calculated from the gradient of the simulated MSD with time, which is roughly 7.24 **×** 10^−8^ cm^2^/s at 300 K. For A' form, the estimated self-diffusion coefficient *D* from the gradient of the simulated MSD is 4.24 × 10^−8^ cm^2^/s at 300 K. However, the variability in the *D* values of A-RNA and A'-RNA seen in LDH is not observed in water. For siRNA, the diffusion coefficients are generally one order smaller than the constrained water molecules in the LDH layers (see Table S1 for the summary of the diffusion coefficients), therefore they are much more stable when complexed with LDH layers. This stability is very important because siRNA could potentially be protected in the delivery processes using nanoparticles as carriers. Of course, once siRNA reaches the target, successful dissociation (release) of siRNA from the hybrid system will become essential. Previous experiments have revealed that strong interaction between carrier and nucleic acid may well hinder the release of the gene from the complex in the cytosol thereby adversely affecting transfection efficiency [60,61]. Fortunately, for MgAl-LDH-siRNA systems, the release of siRNA can be achieved by an acidic environment such as in the lysosomal compartment. The small ions, like Na^+^, K^+^ and Cl^−^, may leave the cell through the so-called ion-tunnels. Therefore, MgAl-LDH seems to have the right balance between chemical stability and biodegradability, and thus very promising for cellular delivery. This is quite different from other gene carriers like polymer-siRNA complex, for which the release of RNA from the complexes may need some specific treatments due to the slow degradation of the delivery system. 

### 3.3. Comparisons with Experiments

**Figure 6:**
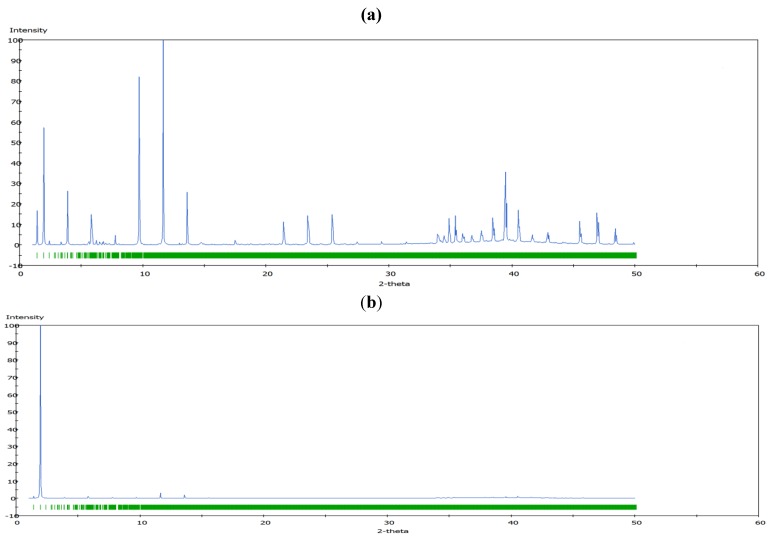
The simulated Powder X-ray diffraction (PXRD) patterns for the LDH + A-RNA hybrid systems using minimized structures: (**a**) partially relaxed LDH + A-RNA; (**b**) fully relaxed LDH + A-RNA.

The calculated powder X-ray diffraction (XRD) patterns of the optimized siRNA-LDH structures are shown in Figure 6 for the A-form (see Figure S9 for A'). Analysis using PXRD enables the determination of highly crystalline materials, usually represented by sharp peaks with higher intensities. Broad XRD peaks would indicate a more disordered layer, and *vice versa*. The figure exhibits characteristic features of a layered structure, e.g., quite pronounced basal reflections (0, 0, 3*n*) due to the strong preferred orientation, and weak broadened non-basal reflections indicating some stacking disorder. 

The diffraction peaks can be indexed based on a hexagonal unit cell with the R
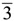
m rhombohedral symmetry generally observed in LDH systems. The peak occurring at a low 2*θ* angle (at about 2°) was attributed to the reflections from the (003) family of crystallographic planes for the central layer. It corresponds to the interlayer repeat distance *d* including a contribution from the metal hydroxide sheet (approximately 0.48 nm) and the interlayer space that contains the guest siRNA anions surrounded by water molecules. The intercalation of siRNA into the LDH was clearly evidenced by the net increase of the interlayer distance, from roughly 0.7 nm (for the two edge layers) to roughly 3.0 nm, for the siRNA layer. The presence of several harmonics of relatively high intensity (especially from the partially relaxed simulations) is evidence of generally well-defined MgAl hybrid materials. Figure 6a also reveals the apparent peaks from two Cl^−^ containing edge layers, which displays a sharp (003) peak at larger angles (at about 10°). In all the cases, the peaks for the central layer at the low angle are the strongest and dominate in the calculated XRD pattern. In the fully relaxed simulations, the corresponding peaks are much lower due to the deformations of the two edge layers (there is a lesser degree of ordering). Since the COMPASS force field is a general *ab initio* force field (*i.e*., not specifically designed for LDH system), the precise degree of deformation of the layers that is observed may vary somewhat from the true situation, but nevertheless the observation is indicative and in accord with findings for other intercalated LDH systems (e.g., [41]). Furthermore, it must be borne in mind that the current periodic simulations correspond to an effective loading of siRNA which may induce greater distortions than the experimental case.

The calculated values of basal spacing for the central layer are summarized in Table S2 from the four sets of MD simulations. It is apparent that the basal spacing between sheets enveloping RNA molecules is enlarged compared to that intercalated with chloride ions only. The principle of setting up our LDH-DNA hybrid system as shown in Figure 1 is that there is some distance between the edges of siRNA and the LDH layers so that some water molecules will be inserted into the empty space. This corresponds to the highly hydrated LDH-RNA systems. Since there are no reported experimental XRPD data for LDH-siRNA systems, our comparisons below will focus on the experimentally studied DNA-LDH systems. Generally speaking, in the experimental co-assembly of DNA with LDH the space between DNA-containing LDH layers is much smaller (e.g., roughly 21.1 Å for the DNA layer in ref [52], see Table S2 for more details). This corresponds to relatively low hydration and in such cases it is also possible that the edge parts of DNA have been partially inserted into the LDH layers or there is some condensation (compaction) of DNA, as pointed out by experiments [53]. Indeed, DNA packaging and condensation is a vital physiological requirement for DNA loading into delivery vectors. This flexibility has also allowed large DNA molecules such as plasmid DNA to be efficiently stored in eukaryotic and prokaryotic cells throughout nature. In this work we have not attempted to explore hydration energies as a function of separation in order to determine an equilibrium interlayer separation, partly because of the time-consuming nature of the simulations and also partly because this should vary depending on the particular experimental case being considered (e.g., crystal powder *versus* in situ aqueous environments). 

## 4. Conclusions

Using molecular dynamics simulations, we have investigated both the structural and dynamical details of the organic-inorganic LDH-siRNA hybrid system. Molecular modeling reveals the arrangement of the guest RNA, layer stacking and spatial distribution of water molecules in the interlayer gallery of the host structure. The present simulations shows that RNA double helices are oriented parallel to the hydroxide layers. Due to strong electrostatic forces acting between the LDH sheets with permanent positive charge and the intercalated RNA, the siRNA molecules have apparent deformations compared to siRNA in bulk water. Meanwhile, the LDH layers also adjust their structure in order to host the siRNA molecules, but the hybrid system still has a well defined layered structure despite the distortions observed. Our work supports the concept that clay-like particles may have acted as hosts which supported and protected diverse RNA structures once formed. These findings support the proposal that the MgAl-LDH host is potentially a good candidate for delivery of functional oligonucleotides such as RNA for gene therapy applications. 

## Supplementary Files

Supplementary File 1 PDF-Document (PDF, 1130 KB)
