# Supplementary material for: Hydrotalcite Intercalated siRNA: Computational Characterization of the Interlayer Environment
Source: Pharmaceutics. 2012 Jun 7;4(2):296–313. doi: 10.3390/pharmaceutics4020296 (PMC3834912; doi:10.3390/pharmaceutics4020296)
Supplement: Supplementary File 1 — PDF-Document (PDF, 1130 KB) [file pharmaceutics-04-00296-s001.pdf]

Supporting Information

Hydrotalcite Intercalated siRNA: Computational Characterization of the Interlayer Environment

Hong Zhang, Defang Ouyang, Vinuthaa Murthy, Yunyi Wong, Zhiping Xu and Sean C. Smith

Figure S1. Optimized structures for the model of A-RNA (a) and A'-RNA (b) using Smart Minimizer in Discover and COMPASS forcefield.

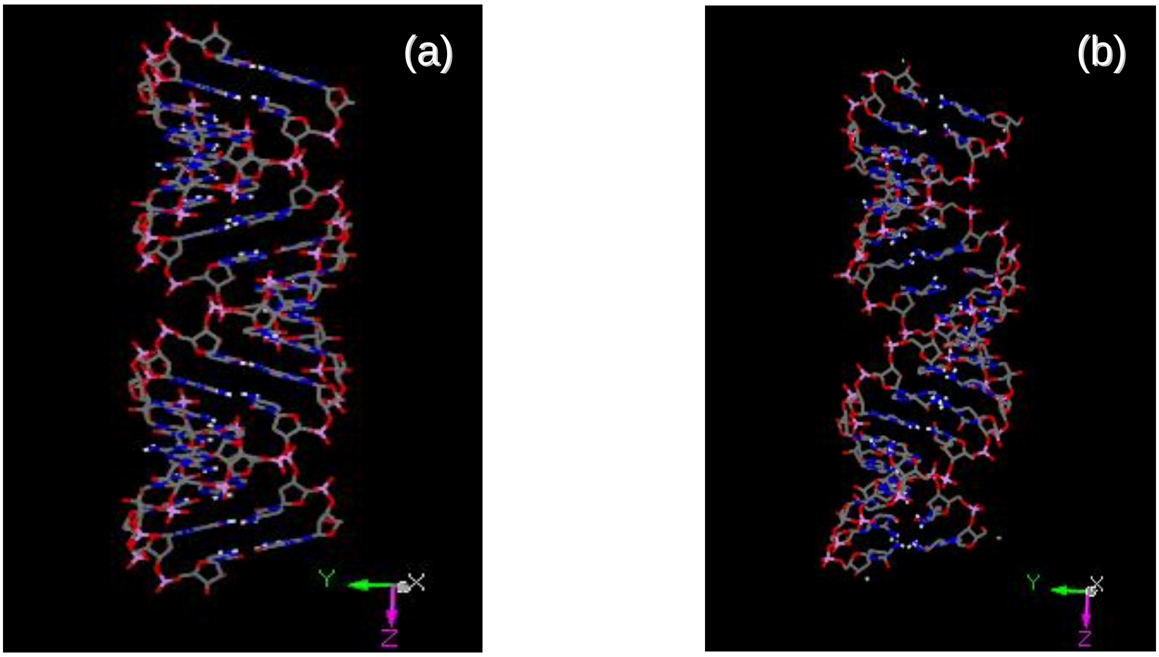

Figure S2. The minimized structures for the partially relaxed LDH hybrid systems of A-RNA (a) and A'-RNA (b). Only siRNA (coloured in yellow for clarity) and water molecules in the central layer are allowed to relax during the initial minimization.

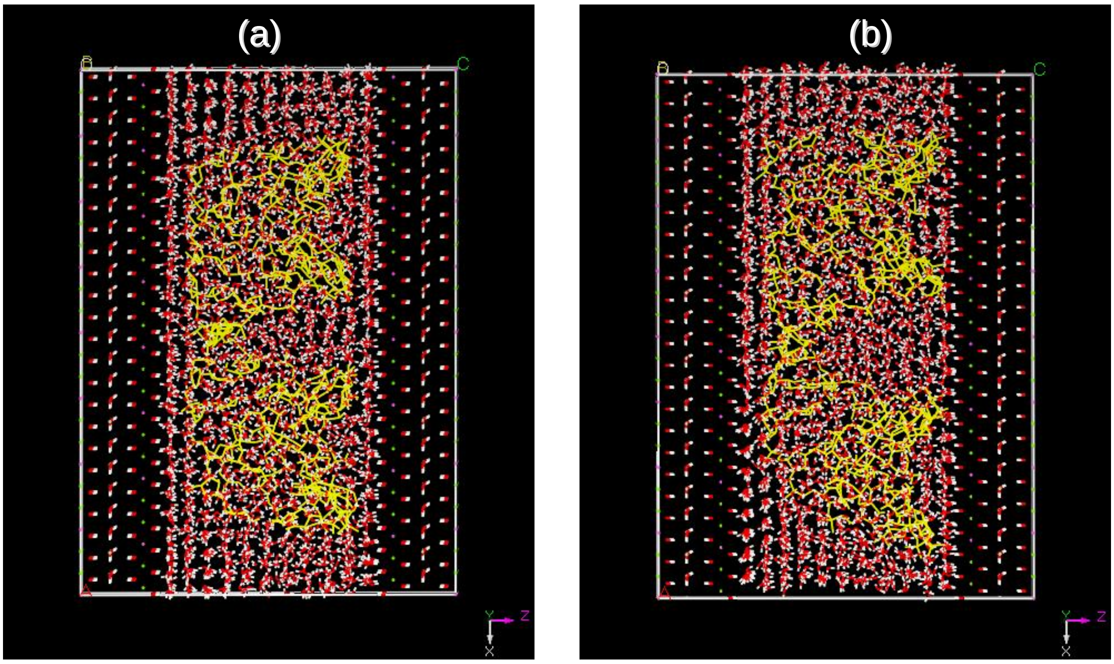

Figure S3. The optimized structures for the fully relaxed hybrid systems of A-RNA (a) and A'-RNA (b). siRNA is colored in yellow for clarity.

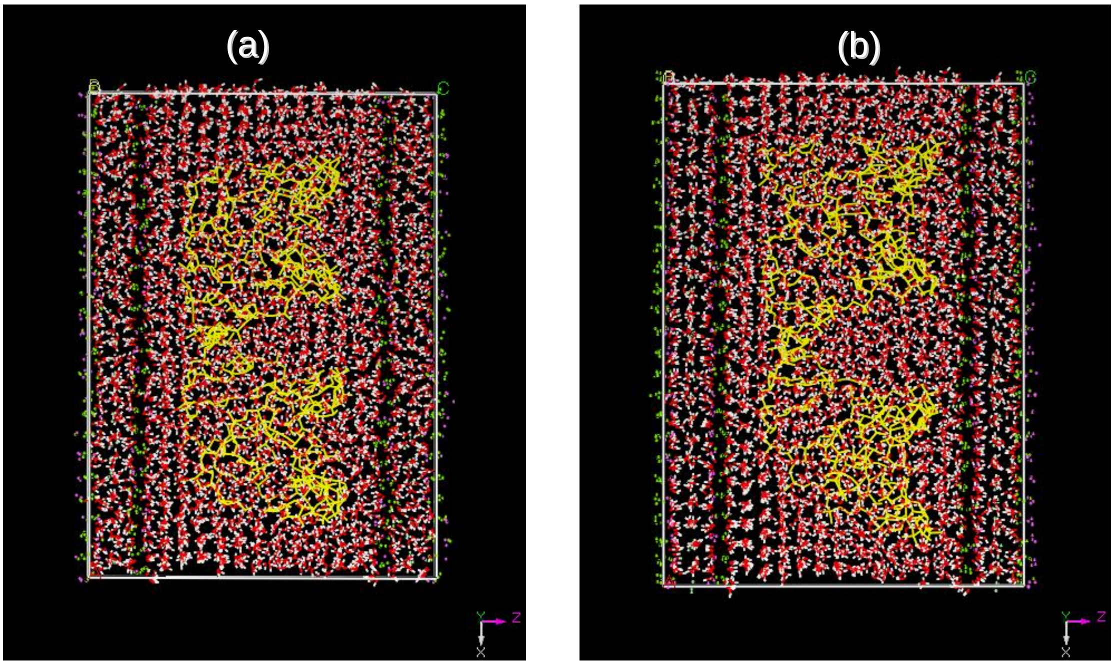

Figure S4. Snapshots at 500 ps for the hybrid LDH + A'-RNA systems from partially constrained simulations in (a) and from unconstrained simulation in (b).

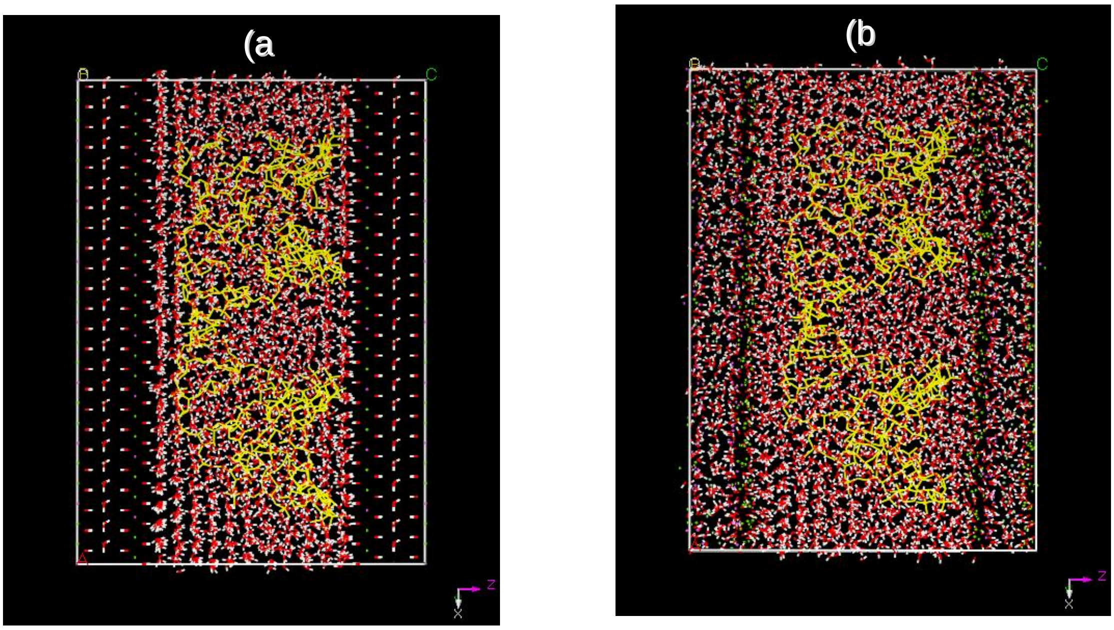

Figure S5. Plots of close contacts for siRNA in the LDH-RNA hybrid systems for A-RNA in (a) and for A’-RNA in (b).

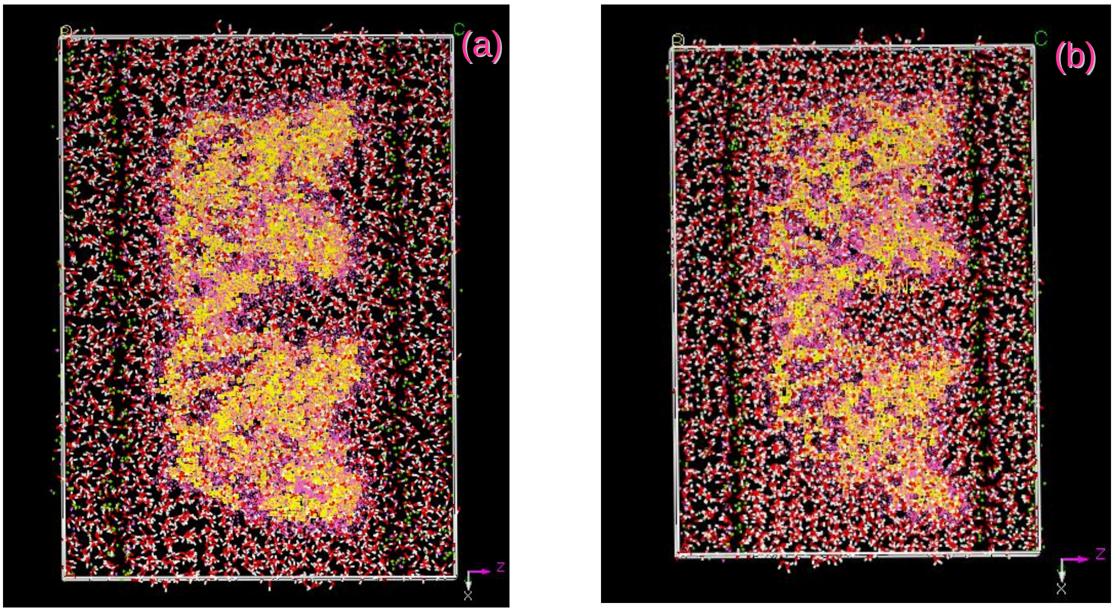

Figure S6. Plots of hydrogen bonding for siRNA in the LDH-RNA hybrid systems for A-RNA in (a) and for A’-RNA in (b).

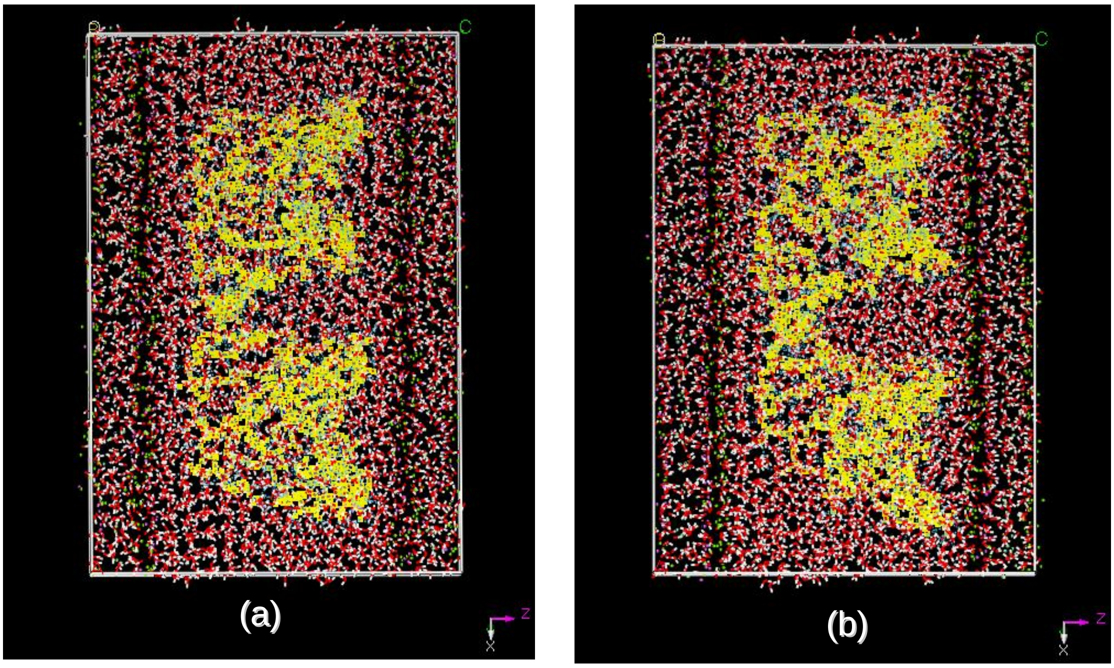

Figure S7. siRNA in water by COMPASS force field in Material Studio 4.4.

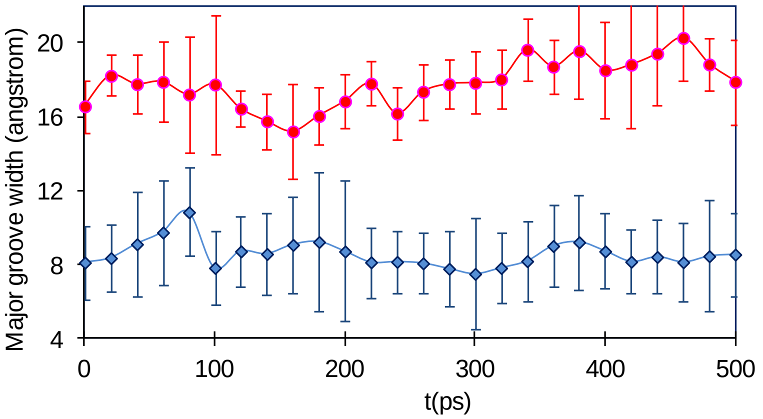

Figure S8. Calculated MSD for water in LDH + A-RNA (a) and LDH + A'-RNA (b) from 500 ps of fully relaxed MD simulations at 300 K.

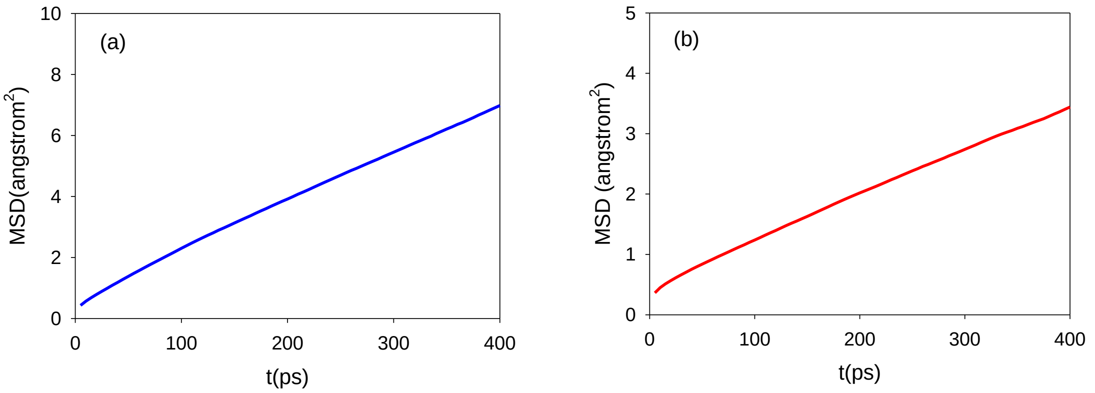

Figure S9. Comparison of PXRD patterns for LDH +DNA/RNA systems from both simulation and experiment. The simulation results (a–b) are from minimized structures of LDH + A'-RNA systems, *i.e.*, (a) for partially relaxed LDH + A'-RNA and (b) for fully relaxed LDH + A'-RNA. The experimental results (c-d) are for $Mg_2AlNO_3^-$ LDHs intercalated with plasmid DNA[5] in (c) and for $Mg_2AlNO_3^-$ LDHs intercalated with linear DNA[6] in (d)

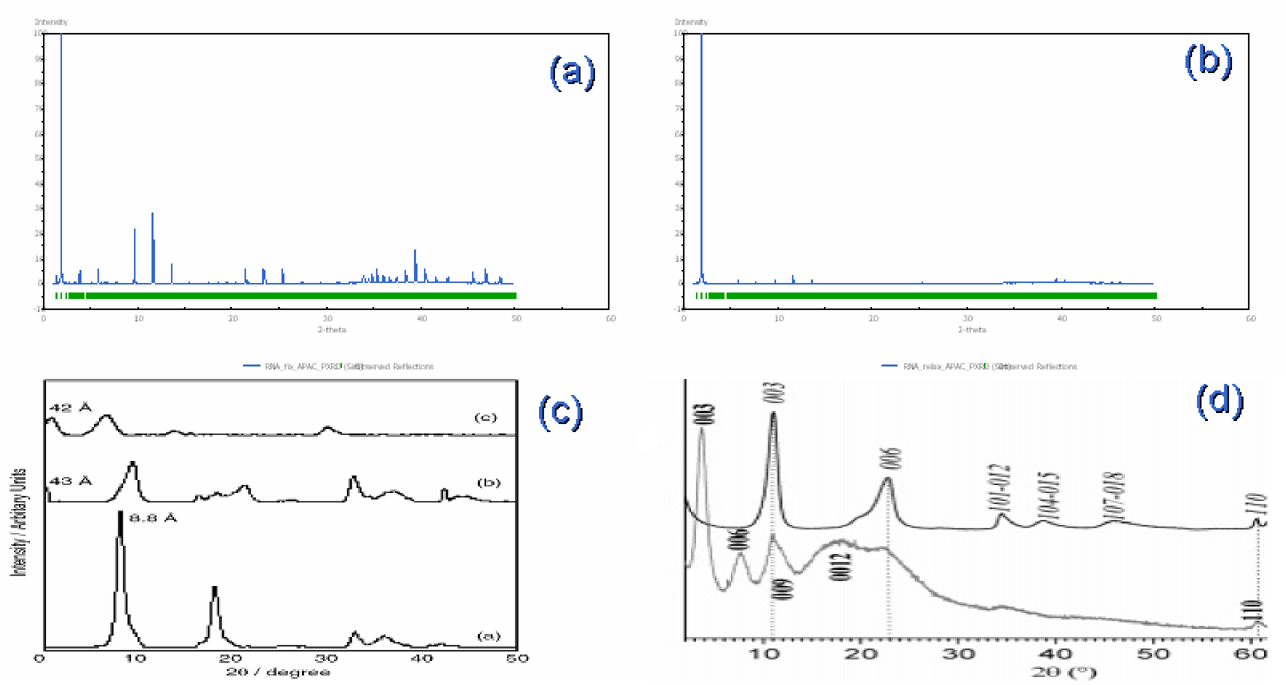

Tables

Table S1. The summary of self diffusion coefficients at 300 K for different LDH intercalates. Unit is in cm^2/s, and * represents this work.

System	*D-water*	*D-siRNA*
A-RNA in water	4.62×10^{-5}	*1.28×10^{-6}*
A'-RNA in water	4.28×10^{-5}	1.61×10^{-6}
LDH + A-RNA*	9.68×10^{-7}	7.24×10^{-8}
LDH + A'-RNA*	4.33×10^{-7}	4.24×10^{-8}
LDH + terephthalate (64 water molecules) [7,27,28]	4.4×10^{-7}	
LDH + terephthalate (44 water molecules) [7,27,28]	1.1×10^{-7}	
Simulations of bulk water [29]	1.88×10^{-5}	
Experimental value for bulk water [30]	2.3×10^{-5}	

Table S2. Comparison of basal spacing from simulation and experiment for LDH + DNA/RNA hybrid systems. Unit is in angstrom, and * represents this work.

System	*d-simulation*	*d-experiment*
LDH + A-RNA (partially relaxed model)*	30.3	
LDH + A-RNA (fully relaxed model)*	32.0	
LDH + A'-RNA (partially relaxed model)*	30.3	
LDH + A'-RNA (fully relaxed model)*	29.9	
LDH/DDNAS [5]		21.1
LDH/DDNAS [1]		23.9
LDH/circular DNA [6]		42.0
